# Lipoprotein(a)-hyperlipoproteinemia as cause of chronic spinal cord ischemia resulting in progressive myelopathy – successful treatment with lipoprotein apheresis

**DOI:** 10.1007/s11789-017-0081-4

**Published:** 2017-02-03

**Authors:** Franz Heigl, Reinhard Hettich, Erich Mauch, Reinhard Klingel, Cordula Fassbender

**Affiliations:** 1 Medical Care Center Kempten-Allgäu, Kempten, Germany; 2 Clinic for Neurology Dietenbronn, Academic Hospital of the University of Ulm, Schwendi, Germany; 3 Apheresis Research Institute, Cologne, Germany; 4 1st Department Internal Medicine, University of Mainz, Mainz, Germany

**Keywords:** Lipoprotein apheresis, Lipoprotein (a), Cardiovascular disease, Spinal cord ischemia, Vascular myelopathy

## Abstract

High concentrations of lipoprotein(a) (Lp(a)) represent an important independent and causal risk factor associated with adverse outcome in atherosclerotic cardiovascular disease (CVD). Effective Lp(a) lowering drug treatment is not available. Lipoprotein apheresis (LA) has been proven to prevent cardiovascular events in patients with Lp(a)-hyperlipoproteinemia (Lp(a)-HLP) and progressive CVD. Here we present the course of a male patient with established peripheral arterial occlusive disease (PAOD) at the early age of 41 and coronary artery disease (CAD), who during follow-up developed over 2 years a progressive syndrome of cerebellar and spinal cord deficits against the background of multifactorial cardiovascular risk including positive family history of CVD. Spastic tetraplegia and dependency on wheel chair and nursing care represented the nadir of neurological deficits. All conventional risk factors including LDL-cholesterol had already been treated and after exclusion of other causes, genetically determined Lp(a)-HLP was considered as the major underlying etiologic factor of ischemic vascular disease in this patient including spinal cord ischemia with vascular myelopathy. Treatment with an intensive regimen of chronic LA over 4.5 years now was successful to stabilize PAOD and CAD and led to very impressive neurologic and overall physical rehabilitation and improvement of quality of life.

Measurement of Lp(a) concentration must be recommended to assess individual cardiovascular risk. Extracorporeal clearance of Lp(a) by LA should be considered as treatment option for select patients with progressive Lp(a)-associated ischemic syndromes.

## Background

Lipoprotein(a) (Lp(a)) was first described in 1963, however, it lasted until 2010 to become fully clear that high Lp(a) concentrations represent an important independent and causal risk factor associated with adverse outcome in atherosclerotic cardiovascular disease (CVD) [1, 2]. A stepwise increase in risk of myocardial infarction was demonstrated with increasing levels of Lp(a) in the Copenhagen city heart study, in particular in case of concomitant risk factors like male sex, smoking history, or hypertension [3]. Causal relationship has been confirmed for peripheral arterial occlusive disease (PAOD) [4]. Lp(a) concentration is also associated with the risk of cerebrovascular ischemia in a curvilinear fashion although less distinct than for coronary artery disease (CAD) [5]. Medical interest is still limited compared to LDL-cholesterol (LDL-C) as high Lp(a) concentrations cannot be effectively corrected by diet or lipid lowering drugs.

Lp(a) consists of a LDL particle and apolipoprotein(a) (apo(a)). It is synthesized in the liver, the physiological function is still unclear [1]. A characteristic of Lp(a) is the more than 1000-fold range of plasma concentrations between individuals from less than 0.1 mg/dl to more than 300 mg/dl with a skewed distribution in most populations [1]. Lp(a) concentrations are under strict genetic control by the LPA gene locus and here especially by a size polymorphism of apo(a) caused by a variable number of kringle IV (KIV) repeats in the LPA gene. The high homology of apo(a) and plasminogen also pointed to the fibrinolytic system, and it was suggested that Lp(a) may act as a modulator of the balance between coagulation and fibrinolysis, and high levels might exert thrombogenic effects.

Lipoprotein apheresis (LA) is the ultimate escalating option to lower blood LDL-C levels in severe hypercholesterolemia. Since 2008 Lp(a)-hyperlipoproteinemia (Lp(a)-HLP) has been implemented in guidelines of statutory health insurance funds in Germany as separate indication for LA. Candidate patients must have Lp(a) levels >60 mg/dl along with progressive CVD despite effective treatment of all other concomitant cardiovascular risk factors in particular LDL-C [6]. The prospective Pro(a)LiFe study demonstrated in patients with Lp(a)-HLP and progressive CVD that prevention of cardiovascular events is a rapid and lasting effect of LA [7, 8].

Here we describe the case of a male patient beginning at the early age of 41 with presentation of established PAOD, who developed a multilocular ischemic syndrome with CAD, cerebellar ischemia and chronic spinal cord ischemia resulting in vascular myelopathy.

## Case report

**Fig. 1:**
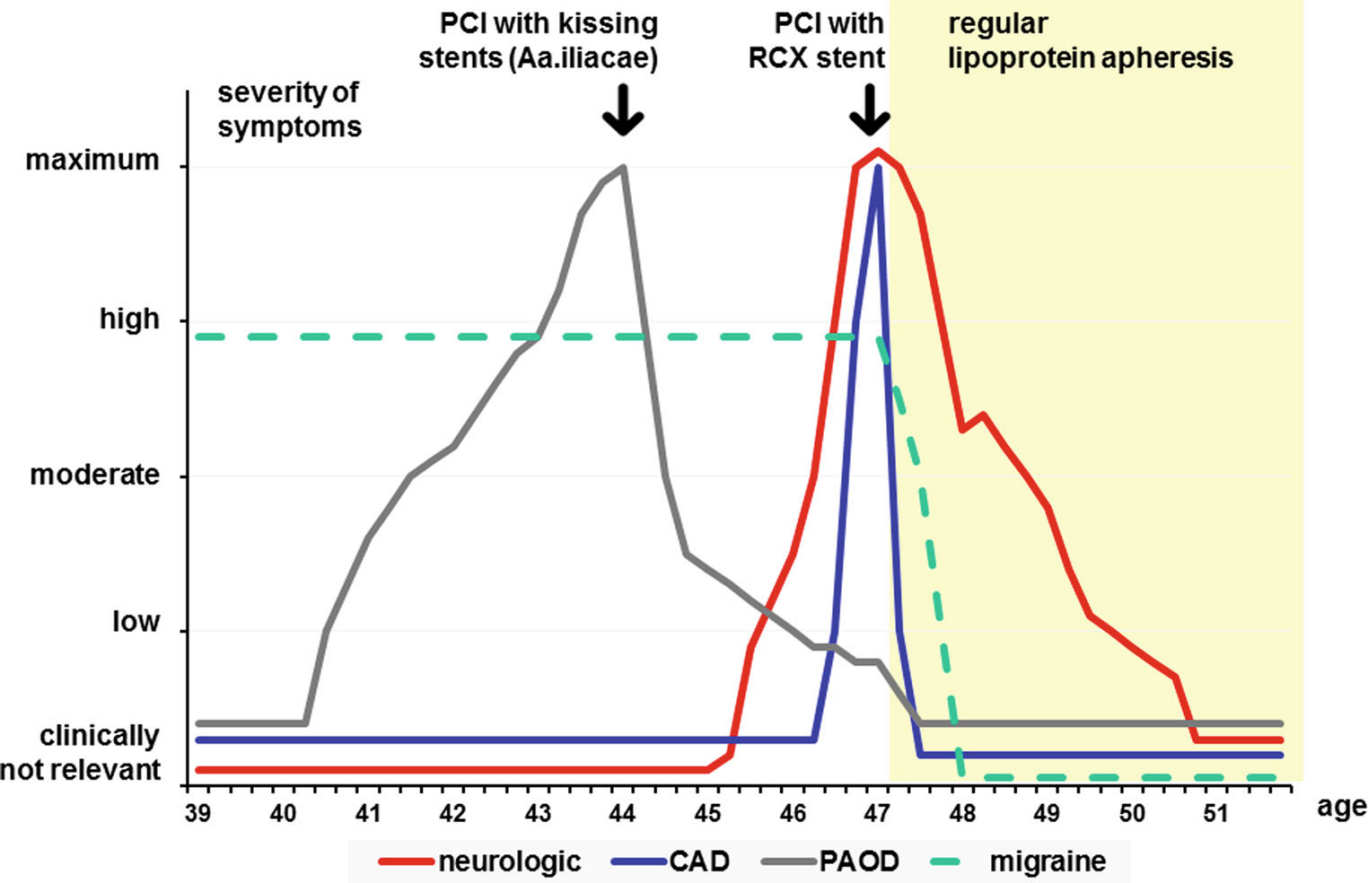
Severity of symptoms related to peripheral arterial occlusive disease (PAOD), coronary artery disease (CAD), or neurological deficits in chronological order. Severity was categorized qualitatively as composite parameter of clinical, or imaging findings, and subjective global assessment of the patient as “clinically not relevant”, “low”, “moderate”, “high”, or at the “maximum” of the particular period of disease

In the following the course of PAOD, CAD and the complex neurological syndrome are described in chronological order (Fig. 1).

## PAOD and CAD

Symptoms of PAOD became clinically significant at the age of 41. The patient experienced exercise-induced pain in his left calf extending up to the thigh and rapidly reaching Fontaine stage IIa. Preexisting and transiently recurring symptoms from lumbar disc lesions made it difficult to determine the exact onset and course of PAOD symptoms. About 3 years later at the age of 44 PAOD had progressed to Fontaine stage IIb with a walking distance less than 50 m. Magnetic resonance imaging (MRI) angiography documented extensive atherosclerosis in both legs with occlusion of the left A. iliaca communis and severe stenosis on the right side. Collateral vessels provided supply of distal arteries in both legs, which did not show stenotic lesions requiring intervention. Successful bilateral percutaneous intervention (PCI) with kissing stent implantation was performed at both Aa. iliacae communes. Blood pressure in the left A. fibularis, which was not detectable before stenting, increased to 100 mm Hg thereafter. The walking distance immediately increased to >200 m and after several weeks the situation improved close to Fontaine stage I. Additionally, diet and treatment of arterial hypertension was optimized at that time. Lipid lowering medication with a statin was supplemented with ezetimibe. LDL-C was 143 mg/dl under simvastatin alone and could be further reduced to 79 mg/dl. At the age of 46 for the first time angina appeared. Single-vessel CAD was documented by coronary angiography with an 80% RCX stenosis. Additional atherosclerotic changes in RIVA and RCA were not stenotic. PCI achieved successful stenting of the RCX stenosis.

## Syndrome of the central nervous system (CNS) involving cerebellum and cervical spinal cord

Since the age of 20 the patient suffered from severe migraine attacks including auras with visual disturbance, which lasted for 2–3 days and occurred usually with intervals of two weeks. At the age of 45 tingling paresthesias developed symmetrically involving lower trunk areas and both legs. One year later paresthesias had spread to the upper back and both arms. Another 3 months later, along with additional sensory impairment of vibration and tactile discrimination, first symptoms of palsy became apparent in legs and arms resulting in complete disability to work and increasing nursing care dependency even including food intake. Over the next 7 months the neurologic syndrome progressed with walking ataxia to spastic tetraplegia, paresthesia, impairment of temperature sensation and the patient became fully dependent on a wheel chair. An extensive diagnostic work-up including MRI of brain and entire spinal cord revealed no indication of ischemic CNS infarction, a demyelinating disorder of the CNS, e. g. multiple sclerosis, any inflammatory or neoplastic disease of the CNS. Neither could imaging of the complete vertebral column detect significant degenerative lesions explaining clinical symptoms.

At the age of 47 progression of bilateral carotid atherosclerosis was documented by ultrasound with increasing intima-media thickness and detection of novel non-stenotic plaque lesions. At the same time the complex neurovascular syndrome extended with substantial neurocognitive impairment, i. e., difficulties to concentrate and loss of memory, vertigo with nausea and vomiting, diplopia and partial vision field loss in the left eye.

According to this complex neurologic syndrome with exclusion of demyelinating, inflammatory, or neoplastic disorders, ischemic vascular disease was hypothesized mainly localized in cerebellum and cervical spinal cord affecting territories of cerebellar arteries downstream of A. basilaris and spinal cord arteries downstream of both Aa. vertebrales, especially A. spinalis ant., A. spinalis post., as well as supply regions of both carotid arteries.

## Diagnosis

The patient exhibited a multifactorial cardiovascular risk profile. He had a history of smoking until the age of 40 with the extent of 20 pack years. Arterial hypertension had been well treated for many years. Hypercholesterolemia was treated with a statin and since the age of 44 LDL-C concentration was below 100 mg/dl. A positive family history of cardiovascular complications was obvious: his mother suffered myocardial infarction at the age of 37 and sudden cardiac death occurred 10 years later, his sister had a non-fatal myocardial infarction at the age of 45, his maternal grandfather had a fatal myocardial infarction at the age of 59 years, hypercholesterolemia was diagnosed in his 22-year-old daughter. The patient had first diagnosis of mild diabetes mellitus at the age of 47 years. Renal function remained stable throughout the entire clinical course. Finally, Lp(a)-HLP was diagnosed with a Lp(a)-concentration of 165 mg/dl. Taking this finding into account, LDL-C corrected for Lp(a)-related cholesterol was below 50 mg/dl, reflecting treatable LDL-C. Considering this risk profile in combination with the clinical course and exclusion of other potential vascular diagnoses, in particular coagulation disorders, embolic disease including cholesterol crystal embolism, autoimmune vasculitis including thrombangiitis obliterans, or thrombotic microangiopathy, Lp(a)-HLP was hypothesized as major underlying etiologic factor. An ischemic pathogenesis due to Lp(a)-HLP was finally diagnosed for the progressive development of neurological deficits. Angiographic verification of spinal cord ischemia as the cause of vascular myelopathy was set aside due to potential complications. Indication for LA was approved by the committee of the regional association of statutory health insurance physicians.

## Clinical course of the patient with regular lipoprotein apheresis

Regular LA was initiated at the age of 47. Due to the severe condition of the patient and in order to reduce Lp(a) concentration to a nearly normal level LA was performed twice a week. Temperature optimized double filtration plasmapheresis (lipid filtration with plasma separator OP-08 W and plasma filter EC-50 W, both Asahi Kasei Medical, Japan, in combination with Octo Nova machine technology, Diamed, Germany), was chosen as LA method. DFPP is one of the fibrinogen eliminating LA methods resulting in an additional immediate effect of rheologic improvement. An arteriovenous fistula was necessary as vascular access. With this regimen average concentrations between consecutive LA treatments were 65 mg/dl for LDL-C and 50 mg/dl for Lp(a).

Two months after commencing regular LA treatment neurological symptoms began to ameliorate and continuously declined with short undulant episodes of cerebellar symptoms. After 4 months short walking was possible using walking frames. Ataxia, visual disturbances, and cognitive impairment improved. After 1 year with regular LA combined with intensive physiotherapy, walking was possible using a walking stick, paresthesias disappeared, cognitive impairment was minimal, and the patient started his work again with several hours per day. Less than 2 years after commencing regular LA, walking was possible without any support for short distances and the patient completed full-time office work. Neurologic rehabilitation further improved after 3 years of regular LA. Vertigo became very infrequent. Walking for 2 h and light gardening work became possible. The most recent status appr. 4.5 years with regular LA showed an almost fully rehabilitated patient regarding neurological symptoms including visual function and completely restored neurocognitive and physical abilities. Interestingly, his migraine attacks had also disappeared completely already few weeks after LA initiation and have not recurred so far. No further progression of atherosclerotic lesions was observed in coronary, peripheral, or CNS vascular beds clinically (e. g. no angina during treadmill exercise test up to 150 watts, unlimited walking distance without claudication), and by ultrasound and MRI imaging.

## Discussion

In summary, we present a male patient with an extraordinary history of multilocular vascular complications beginning at the early age of 41 with PAOD, followed by CAD, and neurological deficits of the CNS in cerebellum and cervical spinal cord. CNS ischemia developed in a chronic fashion over more than 2 years without structural infarction. Cardiovascular risk in this patient had a multifactorial background with the major genetically determined component of Lp(a)-HLP.

Atherosclerotic changes in the arteries supplying the spinal cord are difficult to diagnose because spinal angiography is not a routine diagnostic examination. An atherosclerotic origin can be regarded as reasonable if cerebral, coronary, or peripheral arteries are affected and in particular if there are comorbid vascular risk factors like hypertension, smoking history, hyperlipidemia or diabetes [9, 10]. Several other causes are implicated in ischemia of the spinal cord, which were unlikely to play a role in the presented case: thoraco-abdominal aneurysms, aortic surgery, trauma, history of atrial fibrillation, embolic disease, previous cerebral infarction, aortic dissection, hypotension, spinal arteriovenous malformations, tumors, vasculitis, infection, decompression sickness, coagulopathies, cocaine, sickle cell disease, or degenerative changes like compression of spinal arteries by spondylotic vertebral spurs [9–11]. In 7 to 36.1% the cause remains undefined. The onset of spinal cord infarction is typically abrupt, with maximal symptomatology reached within 72 h for most patients, but also chronic vascular myelopathy can develop [12]. In the case presented here the ischemic spinal cord syndrome exhibited a chronic progressive course over a period of 2 years, without precipitating as structural infarction in any of the affected vascular territories of the CNS. MRI can detect the presence, location and extension of the infarction, but imaging of chronic ischemia without structural infarction is not possible. Preservation of spinal cord function, at least to some extent, is essential for the recovery process due to the high vulnerability of spinal cord neurons. The pace and extent of functional recovery varies. While in most patients who regained function the improvement was already noticed in the first 6 months, gradual recovery often continued to occur over a much longer time. Severe impairment at nadir is the strongest predictor of poor functional outcome. It is very uncommon that wheel-chair dependent patients with a non-traumatic spinal cord ischemic syndrome reach an essentially full functional rehabilitation on follow-up as in this case [13].

The immediate effect of regular LA is pulsed physical extracorporeal elimination of apoB-containing lipoproteins including Lp(a) with its load of oxidized phospholipids [14]. High Lp(a) levels are associated with endothelial dysfunction [15]. A single LA treatment improves endothelium-dependent vasodilation [16] and the elimination of oxidized Lp(a) might be more important to this effect than oxidized LDL [17]. Regarding corrected LDL-C this patient had achieved low levels 3 years before commencing chronic LA suggesting that the cardiovascular benefit of LA substantially derived from the additional elimination of Lp(a) particles. Reduction of plasma viscosity by additional lowering of fibrinogen and other factors of blood coagulation system with subsequent improvement of microcirculatory impairment potentially contributed to the overall therapeutic effects seen in this case. Ruptured plaques tend to have large lipid cores. Improving plaque morphology could be one underlying mechanism of action for preventing cardiovascular events by LA. It was shown that LA quantitatively reduced the number of vulnerable plaques and qualitatively limited the propensity of plaques to rupture and their thrombogenicity [18, 19].

Migraine is a very common neurological disorder, affecting 10% to 15% of the adult population [20]. An association between migraine and vascular diseases has been discussed [20]. It was suggested that the risk of ischemic stroke increases along with the increase in frequency of migraine attacks [21]. A potential link between the long-standing migraine history in this case and development of cerebellar and spinal cord ischemia might be speculated, as for both LA treatment provided therapeutic benefit.

## Conclusion

Genetically determined Lp(a)-HLP must be considered as the major etiological factor of cardiovascular risk in this patient. Treatment with an intensive regimen of regular LA was successful to stabilize the progressive course of ischemic events and to induce very impressive neurologic and overall physical rehabilitation. Regular LA reverted an accelerated progressive course of Lp(a) associated CVD to a stable course in terms of the resolution of neurological deficits, prevention of further events and improvement of quality of life. Measurement of Lp(a) concentration must be recommended to assess individual cardiovascular risk and extracorporeal clearance of Lp(a) by LA should be considered as treatment option for select patients with uncommon ischemic syndromes.
